# Chimeric Antigen Receptor T Cell Therapy Management and Safety: A Practical Tool From a Multidisciplinary Team Perspective

**DOI:** 10.3389/fonc.2021.636068

**Published:** 2021-03-11

**Authors:** María Belen Marzal-Alfaro, Vicente Escudero-Vilaplana, Jose Luis Revuelta-Herrero, Roberto Collado-Borrell, Ana Herranz-Alonso, Maria Sanjurjo-Saez

**Affiliations:** Pharmacy Department, Hospital General Universitario Gregorio Marañón, Instituto de Investigación Sanitaria Gregorio Marañón, Madrid, Spain

**Keywords:** chimeric antigen receptor, pharmacist, pharmacovigilance, practice management, traceability, safety

## Abstract

**Purpose:**

The use process for chimeric antigen receptor T (CAR-T) cell drugs is complex and has been associated with a number of potentially severe complications, which requires management by a multidisciplinary team. Pharmacists are a key element in the team and have roles and responsibilities. Our objective was to develop a structured and practical guide that supports hospital pharmacist responsibilities and defines specific activities in a CAR-T cell therapy program, specifically in Europe.

**Methods:**

A literature review was performed, and the recommendations related to pharmacy practice in CAR-T therapy programs were analyzed. A multidisciplinary team was assembled, and meetings were held to address the key tasks in the CAR-T cells’ management process and to create the guide, based on national and international recommendations and in expert’s opinions.

**Results:**

The multidisciplinary team defined the following key tasks and issued recommendations to improve patient safety, treatment efficacy, and quality: patient selection and evaluation, CAR-T cell drug order to manufacturer, apheresis and material shipment, reception of CAR-T cell drug and storing, CAR-T cell drug prescription and pharmacy verification, CAR-T cell drug thawing and dispensing, CAR-T cell drug administration, patient education, pharmacovigilance and monitoring and outcomes’ record and evaluation. In each task the pharmacist’s role and how it can improve patient care are defined. A checklist was created to guarantee the compliance of standard operating procedures approved in the institution to manage CAR-T cell therapy and as a tool to collect required data for outcomes’ record and evaluation.

**Conclusion:**

This article provides a consensus set of safety recommendations regarding CAR-T therapy management in clinical practice, easily implementable by other institutions in the European setting. The guide identifies key steps where the involvement of hospital pharmacists would improve the safety and quality of the process and is a support guide to standardize hospital pharmacists’ responsibilities within the multidisciplinary team.

## Introduction

Chimeric antigen receptor T-cell therapies (CAR-T) are a type of advanced therapy based on the use of T lymphocytes to identify and kill tumor cells expressing a specific antigen. This new type of immunotherapy consists of T lymphocytes that are genetically manipulated *ex vivo* to express engineered CARs specific for particular tumor targets. These reprogrammed CAR-T cells are subsequently expanded, selected if necessary, and conditioned to produce the drug that the patient will eventually receive. Tisagenlecleucel (Kymriah^®^, Novartis) and Axicabtagene ciloleucel (Yescarta^®^, Kite Pharma EU) were the first two CAR-T cell drugs authorized by the European Medicines Agency (EMA) and the U.S. Food and Drug Administration (FDA). Both therapies target the B-lymphocyte antigen CD19 and are approved: tisagenlecleucel for pediatric acute lymphoblastic leukemia (ALL) and adult diffuse large B-cell lymphoma subtypes (DLBCL), and axicabtagene ciloleucel for DLBCL (1, 2).

The manufacturing and use process for CAR-T cell drugs are complex and highly specialized. The two available CAR-T cell drugs use autologous T cells, which are collected in a leukapheresis process, sent to the manufacturer to be genetically modified to express the CAR and sent back to the treatment center to be reinfused into the patient, after they have received a lymphodepleting chemotherapy regimen (3).

CAR-T cell therapy has been associated with a number of potentially severe complications, such as cytokine release syndrome (CRS) and the immune effector cell-associated neurotoxicity syndrome (ICANS), which require coordinated management by a diverse multidisciplinary team (hematologists, intensivists, neurologists and pharmacists) (4, 5). CRS is a systemic inflammatory response caused by cytokines released by infused CAR-T cells and can lead to widespread reversible organ dysfunction. Manifestations of CRS include fevers, nausea, headache, myalgia, tachycardia hypotension, hypoxia, and organ dysfunction, cytopenias, coagulopathy, and hemophagocytic lymphohistiocytosis. In most severe cases, hypotension requiring high-dose vasopressors and hypoxia requiring mechanical ventilation may occur as life-threating complications (6). Neurologic toxicities are diverse and include encephalopathy, cognitive defects, dysphasias, seizures, and cerebral edema (6). Oncology pharmacists are familiar with this type of toxicities as they are similar to those described with anti-checkpoint immunotherapies (7) or blinatumomab treatment (a bi-specific antibody that targets CD19 and can cause CRS and global encephalopathy) (6, 8).

The implementation of a CAR-T cell therapy program in clinical practice requires an exhaustive preparation of the institution, to accomplish accreditation requirements and the participation of professionals with different backgrounds, in order to guarantee the safe and efficient use of these medications (9). This complex care process has similarities to the logistics of hematopoietic stem cell transplantation (HSCT) (10).

The roles and competencies of the clinical pharmacist involved in HSCT have been defined by several organizations. The American and European societies of Blood and Bone Marrow Transplantation (ASBMT and EBMT) have published recommendations of HSCT clinical pharmacists’ activities and responsibilities (11–13). Both documents endorse pharmacist roles with medication management, patient care, transitions of care, education, and research. Unfortunately, there is little data about the pharmacist’s responsibilities to patients treated with CAR-T cell therapy. The EBMT consensus includes the pharmacist role with managing advanced therapy medicinal products, such as CAR-T cells, as they are licensed as medicines (12). The Foundation for the Accreditation of Cellular Therapy–Joint Accreditation Committee ISCT EBMT (FACT–JACIE) standards on immune effector cells therapy recognizes the role of pharmacists in the clinical team and describes the training and knowledge required (14). Some other publications have addressed the roles and responsibilities of hospital pharmacist with CAR-T cell therapy (15, 16). However, as far as we know, there are not many published experiences of groups that have put these recommendations into practice in a CAR-T cell therapy program.

Therefore, the aim of this work is to develop a structured and practical guide that supports hospital pharmacist responsibilities and defines specific activities in a CAR-T cell therapy program, based on national and international recommendations and in expert’s opinions.

## Materials and Methods

This guide, supporting pharmaceutical responsibilities in the management and safety of CAR-T cell therapy, was designed using the nominal group technique (with direct communications between experts and face-to-face discussion). A multidisciplinary team was assembled with hematologists (HSCT and cell therapy specialists) and clinical pharmacists from different backgrounds: clinical oncohematology, traceability and patient safety, and investigational drugs.

The professionals involved are recognized experts in CAR-T cell therapy and belong to the institution’s multidisciplinary Advanced Therapies Unit, which was one of the first accredited in Spain for the treatment of patients with CAR-T cell drugs. The center is JACIE-accredited (17) (Joint Accreditation Committee—European Society for Blood and Marrow Transplantation (EBMT)) and has treated 35 patients with CAR-T cell drugs between 2019 and 2020.

We performed a review of the literature in order to identify the guidelines and recommendations related to pharmacy practice in CAR-T cell therapy programs. A computerized search was carried out on MEDLINE, through PubMed, using the medical subject heading (MeSH) ‘Immunotherapy, Adoptive’ and the terms ‘Pharmacy’, ‘Pharmacist’, ‘Pharmaceutical care’, ‘Pharmacy Service, Hospital’, ‘Guidelines’, ‘Professional Role’, ‘Safety’, ‘Continuity of Patient Care’. Pharmacists conducted the literature search and extraction of relevant titles among articles published in the last 10 years in English, Spanish, or French. References of reviewed articles were also searched for relevant titles. Recommendations by international and national well-known hematology and pharmacy societies were also included: Foundation for the Accreditation of Cellular Therapy (FACT) (14, 18), American Society for Blood and Marrow Transplantation (ASBMT) (11), European Society of Blood and Bone Marrow Transplantation (EBMT) (12), European Hematology Association (EHA) (10), Specialist Pharmacy Service of the United Kingdom National Health System (19), and Spanish Society of Hospital Pharmacy (SEFH) (20). Finally, the research was complemented with the summary of product characteristics of the CAR-T cell drugs approved: tisagenlecleucel (1) and axicabtagene ciloleucel (2).

The Spanish Ministry of Health published the “Boarding plan of advanced therapies in the National Health System (NHS): CAR therapies” (21), which establishes the organizational and care model that aims to guarantee a planned, equitable, safe and efficient use of CAR-T cell drugs within the NHS. These organizational standards were also considered to develop the guide.

To define the variables recorded for outcomes’ evaluation the team considered the “Pharmacoclinical protocol of tisagenlecleucel and axicabtagene ciloleucel use in diffuse large B-cell lymphoma (DLBCL) in the National Health System” (22) and the “Pharmacoclinical protocol of tisagenlecleucel use in B-cell acute lymphoblastic leukemia (ALL) in the National Health System” (23). Those protocols are defined by a national Experts Commission on the use of CAR-T cell drugs. This Commission reviews approved indications for CAR-T cell therapy in general and authorize each particular case to be treated.

With the information gathered, the pharmacists elaborated the guide considering the key tasks in the CAR-T cells management process. In subsequent meetings, the pharmacists presented the guide and a debate allowed the experts to contrast opinions and reach a consensus on the responsibilities and tasks, considering complexity and challenges of safe CAR-T cells use process. Knowledge in clinical practice of health professionals were taken into consideration. After a review process at the institution, this final guidance on hospital pharmacy tasks and responsibilities within the CAR-T cell therapy program was adopted.

## Results

**Figure 1:**
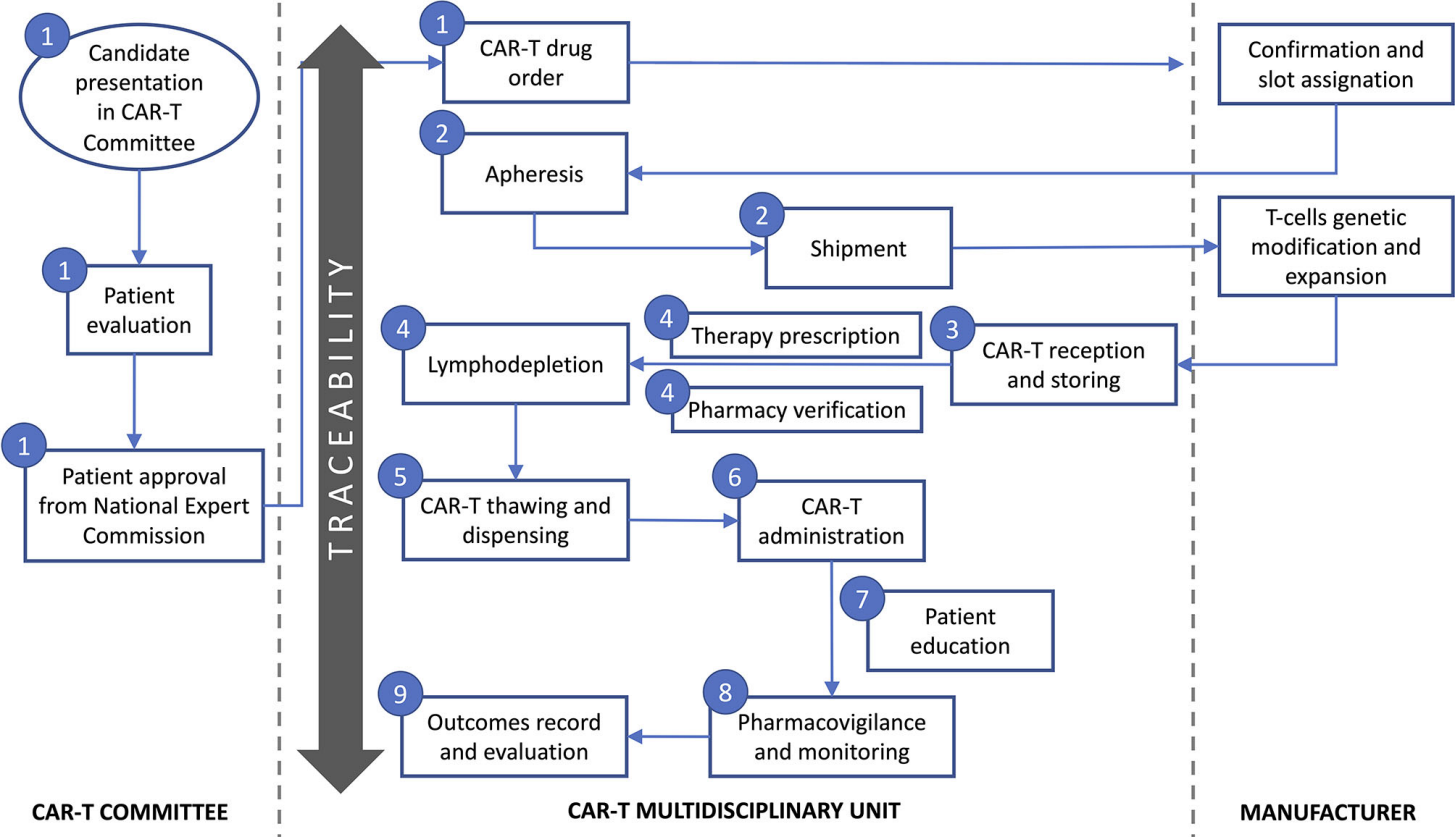
Key tasks during CAR-T cell therapy use process defined by the multidisciplinary team. Numbers represent the guide key points defined in subheading 3 in the text: 1-Patient evaluation and selection and CAR-T cell drug order; 2-Apheresis and material shipment; 3-Reception of CAR-T cell drug and storing; 4-CAR-T cell drug prescription and pharmacy verification of orders; 5-CAR-T cell drug thawing and dispensing; 6-CAR-T cell drug administration; 7-Patient information and education; 8-Pharmacovigilance and monitoring; 9-Outcomes’ record and evaluation.

The multidisciplinary team defined the following key tasks during CAR-T cell drugs use process: patient selection and evaluation, CAR-T order to manufacturer, collection and shipment of leukapheresis product, reception of CAR-T and storing, CAR-T prescription and pharmacy verification of orders, CAR-T thawing and dispensing, CAR-T administration, patient information and education, pharmacovigilance and monitoring, and outcomes record and evaluation (**Figure 1**). Therefore, the guide was structured according to the previously mentioned items. Along with the guide, the team created a checklist that helped with compliance along the processes and as a tool to collect required data to ensure traceability, efficacy and safety (**Table 1**).

**Table 1:** Guide supporting pharmaceutical responsibilities in the management and safety of CAR-T cell drugs.

**PATIENT EVALUATION and SELECTION and CAR-T CELL DRUG ORDER**																																																
Patient Name:																								Date of Birth:																								
Medical Record Number:										National Health System’s identifier:														Manufacturer’s identifier:																								
Manufacturer’s operating system ordering date: _/_/_																								Programmed apheresis date: _/_/_																								
Cell order/Purchase order (Manufacturer):																								Programmed reception date: _/_/_																								
Pharmacy order number:																								Pharmacy order date: _/_/_																								
**APHERESIS and MATERIAL SHIPMENT**																																																
Apheresis Date: _/_/_																								End time:																								
SEC:								DIN Number																																								
E	S																																															
CHECKING STEP																																	CORRECT: YES/NO															
Apheresis prescription Record manufacturer’s identifier and DIN number on prescription (pharmacist)																																	YES/NO YES/NO															
Bag label check: Medical Record Number Name Date of Birth DIN number Manufacturer’s identifier																																	YES/NO YES/NO YES/NO YES/NO YES/NO															
Cryopreservation (if applicable). Conditioning of the apheresis material: Freezing tank temperature: Conservation time:																																																
Apheresis material collection: Date: :/:/: Dewar shipment: YES/NO Vehicle temperature if fresh transport:																																																
**Hematologist signature:**																					**Pharmacist signature:**																											
**RECEPTION of CAR-T CELL THERAPY and STORING**																																																
Received by:																																																
Date and time received: _/_/_ _:_																																																
CHECKING STEP																											CORRECT: YES/NO																					
Packaging integrity visual check																											YES/NO																					
Transit logger temperature checked on receipt (data logger without alarms)																											Temp.:													YES/NO								
Product Identification: Medical Record Number Name Date of Birth DIN number Manufacturer’s identifier Lot number																											YES/NO YES/NO YES/NO YES/NO YES/NO YES/NO																					
Number of bags received																																																
Integrity of the infusion bag(s) visual check																											YES/NO																					
Expiration Date/Correct																																							YES/NO									
Correct storage tank temperature																											YES/NO																					
Receipt documented on manufacturer’s platform																											YES/NO																					
Certificate of Analysis received																											YES/NO																					
Delivery note filed on Pharmacy Department																											YES/NO																					
**Hematologist signature:**																					**Pharmacist signature:**																											
**CAR-T CELL THERAPY PRESCRIPTION**																																																
**Prescribed by (hematologist):**																					**Verified by (pharmacist):**																											
CHECKING STEP																														CORRECT: YES/NO																		
Lymphodepletion regimen order set Patient (Name, Medical Record Number) Medication regimen Dose Supportive medication																														YES/NO YES/NO YES/NO YES/NO																		
CAR-T order set Patient (Name, Medical Record Number) Dose Lymphodepleting therapy interval Supportive medication Record manufacturer’s identifier and DIN number on prescription (pharmacist)																														YES/NO YES/NO YES/NO YES/NO YES/NO																		
Tocilizumab KIT available on ward																														YES/NO																		
**PRODUCT THAW AND DISPENSING**																																																
Date: _/_/_																																																
Thawed by:																																																
CHECKING STEP																																TIME																
Drug removed from storage																																_ _																
Drug received on ward																																_ _																
Thaw process (starts/ends)																																_ _ _ _																
CHECKING STEP																																CORRECT: YES/NO																
Temperature conditions during storage																																YES/NO																
Correct drug Medical Record Number Name Date of Birth Manufacturer’s identifier Lot number Expiration date																																YES/NO YES/NO YES/NO YES/NO YES/NO YES/NO																
Number of bags																																																
Product shipping to ward																																YES/NO																
Thaw process (water bath preparation/sterile conditions)																																Temp.:										YES/NO						
Identification of thawed drug (double label: manufacturer and administration)																																YES/NO																
Administration label number (Needed to correct eMAR registration)																																																
Documentation of the dispensing process in the patient’s electronic medical record																																YES/NO																
**Hematologist signature:**																					**Pharmacist signature:**																											
**CAR-T CELL DRUG ADMINISTRATION**																																																
CHECKING STEP																																CORRECT: YES/NO																
Number of bags administered																																																
Medication administration record by nurse																																YES/NO																
Addition of the tag “PHARMACY –Chimeric antigen receptor” in the patient’s medical record																																YES/NO																
**Pharmacist signature:**																																																

SEC, Single European Code, DIN, Donation Identification Number, eMAR, electronic medication administration record.

From the literature search, the multidisciplinary team selected the most pertinent data to improve patient safety, and treatment efficacy and quality. The team issued the following final recommendations regarding the key tasks:

### Patient Evaluation and Selection and Chimeric Antigen Receptor T Cell Drug Order

The hospital CAR-T Multidisciplinary Committee, integrated by hematologists, oncologists, critical care specialists, immunologists, neurologists, radiologists, hospital pharmacists and nursing staff (21), evaluates candidate patients for CAR-T cell therapy. After patient acceptance, this board coordinates their clinical management in a comprehensive way, before, during and after CAR-T cell therapy (5). As part of this committee, hospital pharmacists check that CAR-T cell drugs are going to be used for an approved indication (10) and according to the therapeutic protocol that defines its use in the NHS (22, 23). They also verify that approval from the Expert Commission on the use of CAR-T cell therapy is in place (15). Moreover, pharmacists may perform medication reconciliation before CAR-T treatment checking patient’s medication list for drug–drug interactions (*e.g.* corticosteroids).

In the European setting, pharmacists have extensive experience in drug acquisition and managing and monitoring the drugs budget. The pharmacy department is in charge of formalizing the purchase order of the advanced therapy medicinal products, as well as billing supervising of these therapies. This responsibility is stated in European legislation (Regulation (EC) No 1394/2007 on advanced therapy medicinal products) (24), that subjects these products to the same principles as other medicines. Pharmaceutical industries have developed platforms for the ordering and management of CAR-T cell therapies. These platforms have been adapted to meet with these criteria, requiring the approval of a hospital pharmacist before the purchasing actually takes place.

Patient identification numbers from hospital and manufacturer are important to be recorded and tracked over the therapy process, and they should be linked to the corresponding product identifiers: manufacturer and pharmacy order numbers. These identifiers are basic to establish a “chain of custody” or “chain of identity” to ensure that every patient receives the correct medicine and that it has been handled properly (25, 26).

### Apheresis and Material Shipment

An apheresis order set was included in the computerized prescription order entry (CPOE) system with two purposes. First, it allows documenting the date of cells’ collection, the manufacturer’s identifiers as well as the Donation Identification Number (DIN), which is an international and unique identifier for a certain collection process. Second, it enables clinical pharmacists to verify that washout periods are fulfilled, as is the case for granulocyte colony stimulating factor (G-CSF), cytotoxics, and steroids (27). From our perspective, the inclusion of the apheresis order in the same system where the CAR-T cell drug prescription and administration are recorded enhances the safety and traceability of the process.

Moreover, during the leukapheresis process, a pharmacist and a hematologist double check the information on the apheresis bag: patient name, medical record number, date of birth, DIN number, and manufacturer’s identifier.

### Reception of Chimeric Antigen Receptor T Cell Drug and Storing

Pharmacists take care of the reception and storage of CAR-T cell drugs, as well as other hospital medications (10, 16). Given that in our institution, the storage is carried out in the Cell Therapy Unit of the Hematology Department, an agreement between the pharmacy and this unit is established to coordinate the reception of the CAR-T cell drug. Manufacturer’s platforms send emails to the reception team, so date and time are arranged in advance.

Pharmacists and hematologists will double check at this step (19): packaging integrity, temperature compliance during transit, integrity of the infusion bag(s), identification and labeling (patient name, medical record number, date of birth, DIN number, manufacturer’s identifier and lot number), number of bags received and expiration date. It is critical that the chain of identity is verified once again at this point (5), so we performed a double-check between the data in the label on the bag and the identifiers we previously recorded.

After reception, pharmacists confirm that the storing conditions are adequate. A continuous temperature monitoring system with 24-h working alarm is in place. Finally, pharmacists document the reception on manufacturer’s platform and file the documentation received, certificate of analysis and delivery note on the Pharmacy Department.

### Chimeric Antigen Receptor T Cell Drug Prescription and Pharmacy Verification of Orders

As part of FACT accreditation, preprinted or electronic orders must be developed for preparative chemotherapy regimens and cell therapy infusion, and must be recorded in chemotherapy administration records (28).

Different lymphodepleting therapy order sets were included in the CPOE system. These order sets are specific for each CAR-T cell drug and diagnostic, and simplify prescription for hematologists. Once the lymphodepleting therapy is prescribed, clinical pharmacists verify the order, focusing on: patient’s identity, expected date of the CAR-T infusion, drug regimen and doses according to the CAR-T cell drug and diagnosis, and supportive drugs (pre-medication, anti-infective prophylaxis). It is our policy not to initiate the lymphodepleting therapy until the CAR-T cell drug of the patient has been received in the hospital and its integrity has been confirmed. Ready to use drugs are compounded in the Pharmacy aseptic service and dispensed to the ward correctly identified to be administered to the patient.

Regarding CAR-T prescription, order sets are developed that include pre-medications like acetaminophen and diphenhydramine to avoid infusion reactions. Before CAR-T preparation and dispensing, pharmacists verify patient identity, lymphodepleting therapy interval and supportive medication. They also verify medication restrictions and washout drug periods prior to CAR-T cell drug infusion (29). Pharmacists record manufacturer’s identifier and DIN number on prescription, in order to guarantee correct traceability.

Pharmacy Service guarantees that a minimum of two doses of tocilizumab are available for each patient receiving CAR-T cell therapy 24/7 (see *Pharmacovigilance and monitoring* for details).

As mentioned, additional order sets were developed for support medications including IV fluids, anti-infective prophylaxis, seizure prophylaxis and anti-emetics. Also, specific order sets were developed for the management of the adverse events such as ICANS and CRS [tocilizumab, siltuximab-unlicensed-, anakinra-unlicensed (30)]. The electronic prescription of every drug needed for the patient allows treatment follow-up by any member of the team in charge of the patient.

### Chimeric Antigen Receptor T Cell Drug Thawing and Dispensing

Thawing and final packing before administration are under the responsibility of a pharmacist (10). In our case, the Hematology and Pharmacy Departments are coordinated for CAR-T cells’ retrieval from the liquid nitrogen tank, transportation on vapor phase dewar to clinical area and defrost procedures (10).

Dispensing procedure requires a double check of the correct drug by a pharmacist and a hematologist when the CAR-T cell drug is retrieved from the storage. The following items must be verified: temperature conditions during storage, patient identity (patient name, medical record number, date of birth), manufacturer’s identifier, lot number, and expiration date. This step is critical to maintain the chain of custody and identity of the CAR-T cell drug. Pharmacists check and record times of each step (removal from storage, reception on ward, start and end of thaw process) to ensure CAR-T cells’ stability from storage to administration to the patient.

Due to the limited stability of CAR-T cells once thawed, in our institution this process is performed in a dedicated area within the HSCT unit. Thawing is done by trained stem cell lab staff, while a pharmacist supervises the procedure checking water bath preparation, temperature, and sterile conditions to prevent medication contamination. This step ends with the attachment of a label to the bag which contains patient’s identification, the DIN code, the manufacturer’s identifier, and a barcode which will be used at bedside by the administering nurse.

The dispensing process is registered by pharmacists in the patient’s electronic medical record to facilitate communication among providers. This documentation is made according to a structured model previously established and includes: drug’s name, DIN number, manufacturer’s identifier, expiration date, infusion date, and number of bags administered.

### Chimeric Antigen Receptor T Cell Drug Administration

As part of FACT accreditation, CAR-T cells’ infusion must be recorded in chemotherapy administration records (28). Administration procedure of the CAR-T cell drug is defined by the multidisciplinary team, and instructions are implemented in the drug label by pharmacists: administration equipment needed, intravenous infusion, infusion rate and perfusion duration, purge and wash technique. Drug labels have a unique barcode which identifies the “five rights” of medication use: the patient, the drug, the time, the dose, and the route (31). Nurses verify the administration in the electronic medication administration record (eMAR) system. In this software, a double check is carried out by reading the barcode of the patient’s identification bracelet and the medication’s label barcode, which includes the administration number. The eMAR allows nurses to record the start and end time of the infusion and any incident that occurs during it.

Once the administration of the CAR-T cell drug has taken place, the pharmacist will tag the patient with “CAR-T CELL THERAPY” in the medical record software. This will allow locating hospitalized patients or admitted to the emergency department who have been treated with CAR-T cells, which will facilitate transitions of care along the hospital and later pharmacovigilance.

### Patient Information and Education

Patients and their families should be given, before being discharged from hospital, appropriate education about early signs and symptoms of CRS and ICANS and their severity to avoid a delay in seeking medical attention (5). Most importantly, patients treated with CAR-T cells should be instructed to immediately alert all providers that they have received this therapy, especially if presenting to a facility outside of their original treatment center (32).

Patients are given an information card for the specific CAR-T cell drug administered, to carry with themselves and present to all healthcare professionals if needed (emergency department, medical visits, pharmacy, *etc*). This card includes:

- Information for the patient: signs and symptoms to go to the emergency department or visit the doctor within the first 8 weeks. For example, temperature monitoring education. Contraindication to driving in the first 8 weeks after CAR-T cell drug administration.
- Information for healthcare professionals: CAR-T cell drug administration date. Contact details of the responsible hematologist, telephone number in case of emergency and contact details of the hospital pharmacist.
- Notice about the precaution of prescribing corticosteroids and contraindication of live vaccines.

The pharmacist is in charge of medication reconciliation at patient discharge. It includes providing medication education to the patient to ensure proper understanding of administration instructions, potential interactions, and adverse effects, as well as we confirmed that patients have been able to get the medicines from their community pharmacy.

### Pharmacovigilance and Monitoring

Health professionals involved in the management of patients treated with CAR-T cells have been trained in the management of the main adverse reactions, in compliance with the FDA-mandated training as part of a Risk Evaluation Mitigation Strategy (REMS) (33, 34). Clinical pharmacists are responsible to ensure that appropriate treatment modalities (*e.g.* tocilizumab) are promptly available for the management of adverse events (10, 19).

As indicated by the FDA (33, 34), the inpatient pharmacy will need to stock at least two doses of tocilizumab for each patient before CAR-T cell administration and ordering protocols and order sets will need to be developed to assure timely administration of both anti-IL-6 therapy as well as corticosteroids and supportive medications as needed for CRS (28). Siltuximab and anakinra are two drugs considered for CRS treatment as second-line, when tocilizumab is not sufficiently effective (30). Both medicines are unlicensed for this indication, so their use has been protocolized in the CRS treatment protocol in our institution.

All the drugs needed for patients treated with CAR-T cells are prescribed in CPOE system by the attending physician and verified by the pharmacist, including medicines for chronic conditions, supportive therapies and drugs for complications or adverse events. Pharmacists create standardized protocols in CPOE for the prescription of tocilizumab, siltuximab, or anakinra with the recommended doses and administration instructions, which will prevent delays in treating adverse events.

The Pharmacy Department compounds tocilizumab and siltuximab for intravenous administration 24 h/7 days a week. To ensure maintenance of adequate stock, tocilizumab, siltuximab, and anakinra vials for CAR-T program are separated from the general store in the pharmacy. Tocilizumab kits have been developed and include four tocilizumab vials of 200 mg, instructions for nurses for its preparation and administration and a withdrawal record sheet. A tocilizumab kit is dispensed to the Marrow Transplant Unit when CAR-T cells’ administration is confirmed, as required by the summary of product characteristics (1, 2). This kit is returned to the pharmacy when the patient is discharged. Clear workflows are developed and coordinated with hematologists and nursing staff for ordering tocilizumab, siltuximab, and anakinra, either during pharmacy working hours or overnight.

Long-term clinical monitoring is important for detection of some complications, such as hypogammaglobulinemia or prolonged cytopenias. Furthermore, the use of a replication­competent viral vector during CAR-T cell manufacturing could pose a theoretical risk to patients of the appearance of secondary malignancies or genotoxicity (35). However, there are currently no studies with a follow-up period long enough to fully characterize this kind of risks. For this reason, a 15 years follow-up period is mandated by the FDA for all such gene therapies (36). In our institution, pharmacists are responsible in the multidisciplinary team to define and coordinate a comprehensive pharmacovigilance CAR-T cell therapy program centralizing the evaluation and reporting of suspected adverse drug reactions to the Spanish Pharmacovigilance System. Long-term toxicity, as well as the risk of genotoxicity or the development of immunogenicity will be evaluated in subsequent Hematology visits and identified by pharmacists by consulting the electronic medical record.

Incorporation of clinical decision support rules might be also beneficial in advancing patient safety and outcomes (37). Due to the known lympholytic effect of corticosteroids, they must be avoided until 3 months after administration, unless no reasonable alternatives exist (29, 38). Exceptions are the use of corticosteroids for the management of CAR-T adverse reactions or life-threatening emergencies (1, 2). Myeloid growth factors, particularly granulocyte macrophage-colony stimulating factor (GM-CSF), have the potential to worsen CRS symptoms and are not recommended during the first 3 weeks after tisagenlecleucel infusion or until CRS has resolved (1, 29). Safety alerts have been included in the clinical decision support system integrated into the CPOE system, such as warnings when corticosteroids or granulocyte colony stimulating factors prescription. The tag “PHARMACY –Chimeric antigen receptor” in the patient’s medical record generates an alert, visible to all health professionals, when the patient arrives at the emergency department or is admitted to hospital. It can avoid prescribing corticosteroids in the emergency department if the patient has been admitted for another reason, or recommend a double verification prior to its use.

### Outcomes’ Record and Evaluation

The registration and analysis of data regarding CAR-T cell therapy, like clinical outcomes, acute complications and long-term toxicities will allow definition of the patients who will benefit the most from these therapies and improve future care. FACT recommends collecting internal data regarding CAR-T cell therapy and submitting them to the Center for International Blood and Marrow Transplant Research (CIBMTR) database for collective analyses (14). In Europe, the EMA requires that all consecutive cases of CAR-T cell therapy be reported to the EBMT registry. Safety and efficacy data should be reported to perform post-authorization studies. In Spain, the “Boarding plan of advanced therapies in the National Health System: CAR therapies” (21) also establishes another information system to report CAR-T cell outcomes in clinical practice (VALTERMED) (39), with the aim of determining the added therapeutic value of CAR-T cell therapies.

**Figure 2:**
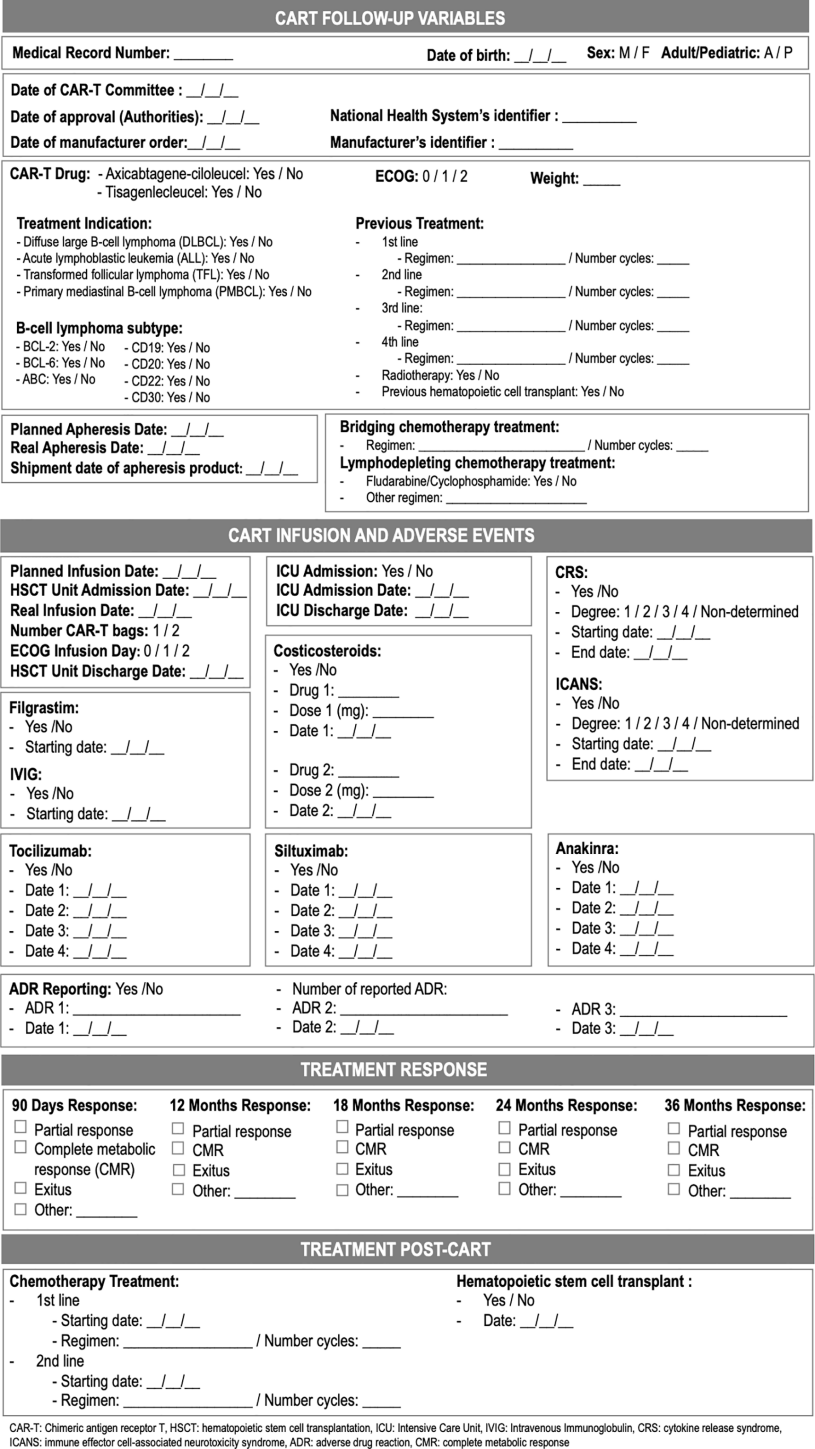
Recorded variables for outcomes’ evaluation.

Although this task is not a pharmacists’ exclusive responsibility, they can collaborate given their extensive experience in clinical informatics and data analysis (40). The CPOE system serves in our model as a repository of therapeutic and traceability data (previous and future therapeutic lines, medicines for chronic conditions, supportive therapies and drugs for complications or adverse events) that can be exploited to describe efficacy and safety of the treatment. The Pharmacy Department designed a checklist to systematically summarize the main outcomes, so it serves as a guide when completing the required databases (**Figure 2**).

## Discussion

CAR-T cells are a new treatment paradigm for malignancies with unmet clinical needs, like B-cell ALL and DLBCL, as they offer a potentially curative option. However, CAR-T cell therapy also requires a complex patient-specific manufacturing and use process and is associated with unique toxicities which need to be recognized and managed. Considered as advanced therapy medicinal products by drug regulatory agencies, hospital pharmacists must be involved in their management to assure an effective and safe use of these medicines.

This article provides a management and safety practice guide for CAR-T cell drug use process and defines the legal responsibilities and the specific activities of pharmacists throughout the process. Since many of these responsibilities are shared with hematologists, the guide was created from a multidisciplinary perspective, in the context of an institutional CAR-T cell therapy program.

Despite the consensus and recommendations issued by professional associations which recognize the role of hospital pharmacists in CAR-T cell therapy programs (12, 16, 20), there is a lack of specific information about how this can be effectively implemented in a daily basis. In a survey that detailed the administrative, logistic, and toxicity management practices on CAR-T cell therapy in the United States, pharmacists were involved in multidisciplinary committees (72% of the respondents), reimbursement process, patient education, and tocilizumab stock management, but only 20% of the respondents have a dedicated CAR-T cell pharmacist (41). This work summarizes the attempt at our institution to identify key steps where the involvement of hospital pharmacists would improve the overall safety of the process. The guide was created by summarizing the most important available evidence found in the literature and has been added to the clinical pharmacist daily practice within the CAR-T cell therapy program.

In particular, this guide is a tool to guarantee the compliance of standard operating procedures approved in the institution to manage CAR-T cell therapy. The guide facilitates the maintenance of the chain of custody and identity of the CAR-T cell drugs and simplifies the checking of all the aspects that guarantee the safety and secure handling of the medicine. Although platforms developed by the pharmaceutical industries are useful tools for tracking the process of shipping and delivery, we strongly recommend that hospitals develop their own records to guarantee traceability throughout the process. The forthcoming approvals of different CAR-T cell drugs from different manufacturers, each one with its own platform, will make difficult to keep traceability in daily practice.

From our point of view, in order to achieve effective implementation of the guide, the coordination of health professionals involved in the CAR-T cell therapy program (hematologists, pharmacists, nurses, and other professionals) is essential, from patient selection to outcome record and evaluation.

As far as we know, there are few published experiences of groups that have implemented pharmacists’ responsibilities in a CAR-T cell therapy program. The Specialist Pharmacy Service of the United Kingdom National Health Service has published a guideline to outline the key areas where chief pharmacists should focus prior to implement CAR-T cell therapy (19). It includes checklists to document key steps, but they comprise a lot of local details and are very extensive, which may be difficult to adopt by other institutions. The Memorial Sloan Kettering Cancer Center has published its process for implementing a commercial CAR-T cell therapy program (28). The authors identified eight essential tasks that define the CAR-T cell workflow and describe clinical, administrative, and regulatory challenges for successful implementation of this program. In this experience, pharmacists are required during infusion and early post-infusion care, to develop order sets for collection and thaw/infusion products, to track and register cell products and to guarantee the availability of tocilizumab. Our guide incorporates all those activities but also includes practical details which may serve as guidance to other institutions to implement pharmacists’ roles and responsibilities.

Pharmacists have a key role as an interdisciplinary team member in CAR-T cell therapy program and their involvement can improve patients’ outcomes, given previous experiences with cancer patients (42–44). Pharmacists are in charge of chemotherapy regimen verification (bridge, lymphodepleting therapy), dose adjustments based on renal and/or hepatic function and review all medications for drug–drug and drug–food interactions. Moreover, they provide comprehensive patient and caregiver education and assess for medication adherence and reconciliation. Pharmacist may find difficulties given some gaps in pharmacy didactic curriculum and experiential education in advanced therapy medicinal products, so this guide may help to implement specific activities throughout the process.

Finally, given the differences in pharmacists’ roles worldwide, this guide is more applicable to the European setting. In Europe, Regulation (EC) No 1394/2007 subjects Advanced Therapy Medicinal Products (ATMPs) to the same principles as other medicines. As these therapies are used in the hospital setting, the hospital pharmacist has the responsibility of contributing to the rational use of CAR-T cell drugs, guaranteeing and assuming the technical responsibility of their selection, ordering, product receipt, storage and preparation, and dispensing (15, 45). Otherwise, some of the processes will not be applicable to centers in United States.

In this scenario, this guide is a practical and comprehensive version of the international recommendations that considers pharmacists’ responsibilities regarding the management and safety of CAR-T cell drugs. Systematic processes with a multidisciplinary approach are useful in health systems. The tool is easy to use and its implementation requires little time from health professionals and minimal technological investment.

## Conclusion

This article provides a consensus set of safety recommendations regarding CAR-T cell therapy management in clinical practice and a practical support guide to standardize hospital pharmacists’ responsibilities within the multidisciplinary team, mainly in the European setting. The proposed guide may help with the compliance of standard operating procedures and to keep traceability throughout the CAR-T cell drug use process in order to guarantee the safe and efficient use of these medications.

## Data Availability Statement

The original contributions presented in the study are included in the article/supplementary material. Further inquiries can be directed to the corresponding author.

## Author Contributions

MM-A, VE-V, JR-H, RC-B, AH-A, and MS-S contributed to the conceptualization and design of the study. MM-A, VE-V, JR-H, and RC-B contributed to the data curation and data analysis. MM-A wrote the first draft of the manuscript. All authors contributed to the article and approved the submitted version.

## Conflict of Interest

The authors declare that the research was conducted in the absence of any commercial or financial relationships that could be construed as a potential conflict of interest.
